# Evaluation of a Novel Tapered Tip EUS-FNB Needle: A UK Multicentre Study

**DOI:** 10.3390/cancers17203390

**Published:** 2025-10-21

**Authors:** Darragh Storan, John Leeds, Arif Hussenbux, Mohamed Elseragy, Ruridh Allen, Tareq El Menabawey, Aaron McGowan, Matthew T. Huggett, Umair Kamran, Bidour Awadelkarim, Beate Haugk, Kofi Oppong, Manu Nayar

**Affiliations:** 1 Freeman Hospital, Newcastle Upon Tyne Hospitals NHS Foundation Trust, Newcastle Upon Tyne NE7 7DN, UKbeate.haugk@nhs.net (B.H.); manu.nayar@nhs.net (M.N.); 2 Population Health Sciences Institute, Newcastle University, Newcastle Upon Tyne NE2 4AX, UK; 3 Leeds Teaching Hospitals NHS Trust, Leeds LS1 3EX, UK; 4 Royal Infirmary of Edinburgh, Edinburgh EH16 4SA, UK; 5 University College London Hospitals NHS Foundation Trust, London NW1 2PG, UK; 6 Translational and Clinical Research Institute, Newcastle University, Newcastle Upon Tyne NE1 7RU, UK

**Keywords:** EUS, FNB, needle, Acquire S, SharkCore, tapered tip

## Abstract

**Introduction**: A new core biopsy needle with a novel tapered stylet tip has been introduced for endoscopic ultrasound-guided fine needle biopsy (EUS-FNB). The tapered point stylet is purported to improve ease of puncture, leading to improved tissue acquisition and accuracy. However, there are no data available in the published literature. The aim of this study was to compare the diagnostic performance of the tapered stylet needle with conventional end-cutting FNB needles for tissue acquisition from solid lesions. **Methods**: Patients who underwent EUS-FNB of a solid lesion using the tapered stylet FNB needle across four tertiary hepatopancreaticobiliary centres in the UK were included in the study. Demographic, clinical, and performance outcomes were included in the analysis. Diagnostic performance was compared with a similar cohort of patients who underwent EUS-FNB using standard end-cutting FNB needles with a blunt-tipped stylet. The primary outcome was accuracy for the diagnosis of malignancy. **Results**: A total of 270 patients were included for analysis; 129 patients (48%) had sampling with the novel tapered stylet tip needle, among which 50% were female, the median age was 69, 74% had pancreatic lesions, and 80% had a final diagnosis of malignancy; 141 control cases (52%) were included for comparison, among which 48% were female, the median age was 68, 67% had pancreatic lesions, and 72% had a final diagnosis of malignancy. The tapered stylet needle demonstrated a sensitivity of 90% and an NPV of 72% for the diagnosis of malignancy compared with 88% and 77% for controls (*p* = 0.147). The overall diagnostic accuracy of the tapered stylet needle was 92.2% compared with 91.5% for controls (*p* = 0.634). **Conclusions**: The novel tapered tip stylet FNB needle demonstrated comparable sensitivity, NPV, and diagnostic accuracy to conventional FNB needles. This is the first and largest study reporting results for this new needle. However, further large comparative studies are warranted to validate our results and to determine if the tapered stylet offers an advantage over the conventional design.

## Simple Summary

Endoscopic ultrasound fine needle biopsy (EUS FNB) is the investigation of choice for pathological diagnosis of pancreaticobiliary solid tumours. In the era of precision oncology, tissue samples of sufficient volume and quality are required to enable next-generation sequencing and ancillary studies. A novel tapered stylet FNB needle has recently been introduced for EUS FNB with the aim of improved tissue acquisition, but to date, there are no comparative data available in the literature. The present study investigated the performance of this new FNB needle across several tertiary HPB centres in the UK. We compared outcomes to a similarly matched cohort that underwent sampling using legacy FNB needles as a control arm. We collected data for 129 patients using the novel tapered tip FNB needle and 141 control cases. Both groups demonstrated a high sensitivity and accuracy for the diagnosis of malignancy, with no significant difference in outcomes. This is the first study we are aware of reporting outcomes for the new tapered stylet FNB needle.

## 1. Introduction

Endoscopic ultrasound-guided tissue acquisition (EUS-TA) has emerged as a cornerstone technique in the diagnostic algorithm for a wide range of gastrointestinal and adjacent solid lesions. Since its introduction in the early 1990s, EUS has evolved from a purely diagnostic imaging modality to a powerful interventional platform, capable of accessing and sampling deep-seated structures with remarkable precision [1,2]. Among its most impactful applications is the acquisition of tissue from pancreatic masses, subepithelial lesions, mediastinal and abdominal lymphadenopathy, adrenal lesions, and hepatic or peritoneal abnormalities [1]. The minimally invasive nature of EUS, combined with real-time imaging and access to previously unreachable anatomical areas, has made it an indispensable tool in modern gastroenterology and oncology practice [2].

For more than two decades, fine-needle aspiration (FNA) was the standard technique for EUS-guided tissue acquisition. FNA allowed for cytologic diagnosis with acceptable sensitivity and specificity for many malignancies, particularly in the pancreas. However, the limitations of FNA became increasingly apparent with the advent of molecular oncology and personalised medicine. Cytologic specimens often lack sufficient cellularity, tissue architecture, or stromal context for accurate histopathologic subclassification, immunohistochemical staining, or molecular profiling [3]. To address these shortcomings, fine-needle biopsy (FNB) needles were developed with the explicit goal of retrieving histologically intact core tissue [3,4]. Unlike FNA needles, which are typically side-cutting or blunt-tipped and optimised for cytology, FNB needles feature advanced tip designs such as Franseen, fork-tip, or reverse bevel to facilitate “end-cutting” and the harvesting of core samples [4,5]. Numerous randomised controlled trials and meta-analyses have now shown that FNB needles not only yield higher rates of diagnostic adequacy and accuracy but also reduce the number of passes required, which may improve procedural efficiency and reduce patient discomfort [6,7,8]. The current generation of end-cutting fine-needle biopsy (FNB) needles has reported accuracy rates of >90% for the diagnosis of malignancy [6,7,8], and they are considered the needles of choice for tissue acquisition from solid lesions. In contrast, fine-needle aspiration (FNA) needles are now reserved for pancreaticobiliary access and pancreatic cyst fluid assessment [8,9].

**Figure 1:**
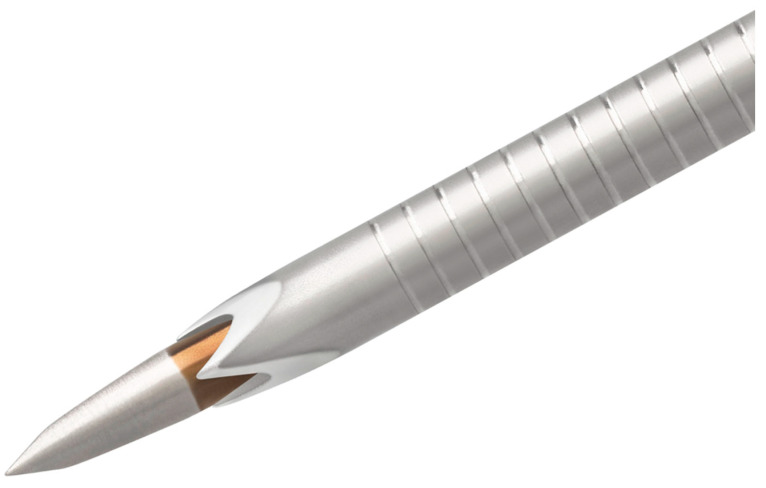
Acquire S FNB needle tip with a tapered stylet (Boston Scientific).

Among the most widely adopted FNB designs are the SharkCore (Medtronic, fork-tip) and Acquire (Boston Scientific, Franseen-tip) needles [9,10,11]. Both have demonstrated excellent performance across a range of indications. However, as the need for high-quality tissue continues to rise, driven by genomic profiling and biomarker-driven therapy selection, efforts are underway to further refine EUS needle technology. A novel advancement in this field is the Acquire S needle, a next-generation FNB needle introduced by Boston Scientific. This device retains the standard Franseen-tip geometry but incorporates a unique tapered stylet, marking a significant departure from conventional blunt-tip stylet designs (Figure 1). The tapered stylet is engineered to remain exposed during initial tissue puncture, rather than being withdrawn prior to insertion. This innovation is hypothesised to reduce tissue resistance and friction at the point of entry and improve penetration into fibrotic or densely vascularised lesions. In theory, these properties could reduce tissue trauma, preserve histologic integrity, and ultimately improve diagnostic performance. 

Despite the theoretical advantages of the tapered stylet design, no published studies to date have evaluated the real-world diagnostic performance of the Acquire S needle. Most of the existing literature on EUS-FNB focuses on comparisons between broad-tip geometries (e.g., Franseen vs. fork-tip) or needle gauges, while few have investigated the impact of internal stylet design on sample quality, diagnostic yield, or operator ergonomics [4,5,8].

The current study was, therefore, undertaken to evaluate the diagnostic performance of the novel Acquire S FNB needle with a tapered stylet compared with standard FNB needles utilising a blunt-tip stylet across a multicentre cohort of patients undergoing EUS-guided sampling of solid lesions. We aimed to assess overall diagnostic accuracy and sensitivity for malignancy. By leveraging real-world data from four high-volume tertiary centres, this study seeks to provide early clinical evidence regarding whether this design innovation translates into improved diagnostic outcomes in routine practice.

## 2. Materials and Methods

### 2.1. Data Collection

**Figure 2:**
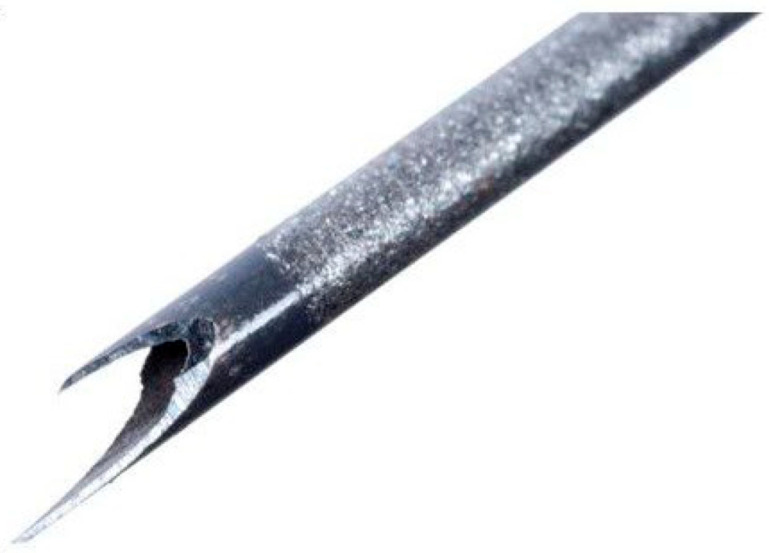
SharkCore FNB needle tip (Medtronic).

**Figure 3:**
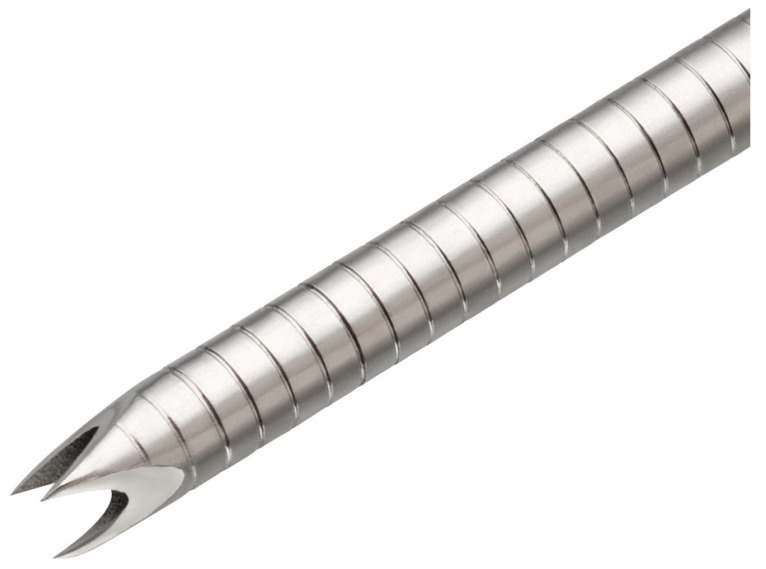
Acquire FNB needle tip (Boston Scientific).

Data were collected prospectively from four tertiary, high-volume hepatopancreaticobiliary (HPB) centres across the UK that each perform > 750 EUS per annum. All of the procedures were carried out by an expert PB endoscopist, while histological assessment was performed by expert pathologists. Data collection commenced in November 2023, at the time the Acquire S FNB needle was introduced to these units, and was completed in August 2024. Consecutive adult patients (≥18 years) attending for EUS-guided sampling of a solid lesion were included for analysis. A similarly matched cohort over the preceding time period prior to the introduction of the Acquire S needle was included as a comparator group. The needles used in the comparator group were the fork-tipped SharkCore FNB needle (Medtronic, Minneapolis, MN, USA) (Figure 2) and the Franseen-tipped Acquire FNB needle (Boston Scientific, Marlborough, MA, USA) (Figure 3), depending on the treating centre’s preference. Both of these needles are end-cutting with a blunt-tipped stylet. The number of passes, needle size, and aspiration technique were at the discretion of the treating endoscopist. Pathologists were not routinely present in the endoscopy room, nor was macroscopic on-site evaluation (MOSE) routinely performed. Patient demographics, lesion size, anatomical location, needle type, needle size, number of passes, tissue adequacy, histological diagnosis, and complication rate were recorded.

### 2.2. Histological Reporting

The final histological diagnoses were classified into 5 categories similar to those used in the reporting of pancreaticobiliary cytology: inadequate, benign, atypical (i.e., non-diagnostic for either benign or malignant disease), suspicious of malignancy, and diagnostic for malignancy. Inadequate samples were defined as those with no or very scanty pancreatic or lesional cell representation insufficient to rely on for diagnostic assessment [11,12].

### 2.3. Definitions and Outcomes

The primary outcome was the diagnostic performance of the tapered tip stylet Acquire S FNB needle for the diagnosis of malignancy as compared with standard blunt-tipped FNB needles. Samples reported as benign or atypical were categorised as negative for malignancy, while samples reported as highly suspicious or malignant were categorised as positive for malignancy. A secondary “stricter” analysis was also performed, which categorised only samples reported as malignant as diagnostic of malignancy. For the purposes of the study, low-grade malignant tumours (i.e., gastrointestinal stromal tumours, lymphoma, and neuroendocrine tumours) were considered malignant. Specimens that contained inadequate material were considered negative for malignancy [12,13].

Diagnostic performance was expressed using standard performance parameters, including sensitivity, specificity, and overall accuracy. The final gold standard diagnosis for malignancy required unequivocal malignant pathology obtained by EUS sampling, surgical resection, or alternative biopsy. For non-operated patients with non-diagnostic tissue sampling, clinical and radiological disease progression consistent with malignancy at 6-month follow-up was required. Benign classification required non-malignant and non-suspicious tissue sampling and a follow-up of at least 6 months with no evidence of malignancy on interval imaging [12,13].

### 2.4. Statistical Analysis

Categorical data were expressed as numbers and percentages, while continuous data were expressed as medians and ranges. Categorical and continuous variables were analysed using the chi-squared test, Student’s *t*-test, Mann–Whitney U test, and one-way ANOVA as appropriate. The area under the receiving operator characteristic curve (ROC) analysis was performed to calculate diagnostic performance outcomes for each needle. A *p*-value of less than 0.05 was considered to be statistically significant. Statistical calculations were performed using IBM SPSS statistics for Macintosh, version 29.0.2.0 (IBM Corp., Armonk, NY, USA).

### 2.5. Study Approval

The study was registered as a clinical audit in line with National Health Service (NHS) guidelines. Data collection was performed as part of an ongoing clinical audit of the quality of our service. Normal NHS Clinical Audit Practice was observed. All aspects of the study were conducted in accordance with the Declaration of Helsinki 1964, as revised in Tokyo 2004. Formal ethical approval was not required. Written informed consent was obtained from all patients prior to the procedure.

## 3. Results

### 3.1. Baseline Characteristics

Data were collected for 277 patients who underwent EUS-TA using either the Acquire S or standard FNB needle. Seven patients did not have a solid mass lesion and were excluded from analysis. A total of 270 patients were included for final analysis, comprising 129 Acquire S cases and 141 controls (94 SharkCore, 47 standard Acquire). Demographic, clinical, and technical characteristics are outlined in Table 1. Both groups were similarly matched, apart from a significantly higher number of biliary tract lesions and a larger median lesion size in the control group. For those with a malignant diagnosis, the final pathological diagnosis was adenocarcinoma for 163 cases (79.5%) and neuroendocrine tumour for 24 cases (11.7%), with the remainder (<10%) made up of GIST, lymphoma, metastatic melanoma, metastatic transitional cell carcinoma, metastatic renal cell carcinoma, hepatocellular carcinoma, squamous cell carcinoma, non-small cell lung cancer, and acinar cell carcinoma, as outlined in Table 1.

### 3.2. Diagnostic Performance

Sample adequacy was high in both groups—95.3% for the Acquire S needle versus 92.9% for controls (*p* = 0.396). The distribution of cytological diagnoses was as follows: inadequate (6.3%), benign (19.6%), atypical (6.3%), highly suspicious for malignancy (5.9%), and malignant (61.9%).

Diagnostic performance metrics for each group are shown in Table 2. The Acquire S needle demonstrated a high sensitivity (90.3%), specificity (100%), accuracy (92.2%), and negative predictive value (NPV) (72.2%). There was no significant difference in performance outcomes when compared with the standard FNB needles. 

To assess robustness, a stricter secondary analysis was performed, classifying only cases with definitive malignant cytology as malignant—excluding those labelled “suspicious”. Under this definition, diagnostic accuracy remained comparable between the groups: 87.6% for the Acquire S needle and 85.8% for standard needles (*p* = 0.667; see Table 3).

### 3.3. Subgroup Analyses

A subgroup analysis focused on patients who underwent sampling of pancreatic solid lesions, as outlined in Table 4. All needle types demonstrated high diagnostic performance for these lesions, with no statistically significant differences in sensitivity or accuracy, with both exceeding 90%.

### 3.4. Adverse Events

No adverse events were reported for either group during the procedure or within 30 days of follow-up.

## 4. Discussion

This multicentre prospective study is the first to evaluate the diagnostic performance of a novel EUS-FNB needle that employs a tapered stylet tip. The tapered stylet tip is designed to facilitate easier puncture of solid lesions, potentially improving diagnostic yield. However, despite these potential advantages, our study found no significant difference in diagnostic performance compared with conventional second-generation end-cutting FNB needles incorporating a standard stylet design.

The tapered stylet needle demonstrated a sensitivity of 90% and an NPV of 72% for the diagnosis of malignancy, closely mirroring the control group’s sensitivity of 88% and NPV of 77%. Similarly, there was no statistical difference in overall diagnostic accuracy, with both groups achieving >90% accuracy. These results are in keeping with previous studies, which have shown high diagnostic accuracy rates for end-cutting FNB needles in the range of 8595–% [5,7,10,14]. These findings indicate that the novel design performs comparably to established needle technologies in real-world clinical settings, particularly within high-volume tertiary hepatopancreaticobiliary centres.

In the era of precision oncology, treatment decisions are increasingly guided by molecular and genetic characterisation of tumours. These approaches require tissue samples of sufficient volume and quality, driving the continued evolution of EUS-FNB technology [3,15,16]. A core tissue with intact histological architecture is the optimal sample for performing molecular profiling [17,18,19]. Tumor surface area and tumor cellularity are essential parameters in determining sample adequacy for molecular testing. Up to 2000 cells and 30% tumor cellularity may be necessary to perform a comprehensive genomic analysis, but analysis can be successful at <10% tumor cellularity [20]. Newer-generation FNB needles—such as those with fork-tip and Franseen designs—have previously demonstrated improved diagnostic yield and histological core acquisition compared to earlier generations and traditional FNA needles [10,13,21]. For genomic profiling of pancreatic lesions, previous studies have demonstrated that specimen adequacy was considerably higher for FNB needles compared with FNA needles [19,21]. The introduction of the tapered tip stylet represents a notable innovation, even though the current study has not demonstrated a clear advantage over conventional FNB needles. Continued research and development in this area remains essential as the diagnostic demands increase with the integration of next-generation sequencing (NGS).

While no significant diagnostic superiority was observed, the potential procedural advantages of the tapered design warrant consideration. The Acquire S needle is designed for smoother lesion entry and potentially less resistance in fibrotic or calcified lesions. These benefits could translate into shorter procedure times or fewer needle passes, particularly in difficult anatomical locations, though these endpoints were not directly assessed. Importantly, while diagnostic yield remains the primary endpoint in most EUS-FNB studies, future relevance increasingly lies in tissue quality. The need for high-quality, architecture-preserving cores is critical for NGS, which is now routinely used in pancreatic adenocarcinoma and other malignancies [18,21,22]. Although this study did not quantify specimen length or suitability for molecular profiling, future investigations should incorporate these endpoints.

This study has several strengths, including its multicentre design, real-world patient population, and the largest sample size to date evaluating this novel needle. The predominance of pancreatic lesions (~70%) and high malignancy rates (>70%) in both cohorts reflects a representative sample of patients referred for EUS-FNB in tertiary hepatopancreaticobiliary centres. Limitations include the study’s non-randomised design and retrospective collection of comparator data, which introduce potential selection and reporting biases. Procedural factors such as the number of passes, needle gauge, sampling technique, or tissue processing techniques were not controlled for, which may have influenced outcomes, although no significant difference in needle size or number of passes was noted between groups [23]. Furthermore, a histological review was performed locally at each centre, introducing potential inter-observer variability. Finally, although diagnostic outcomes were comparable, the tapered stylet design may offer procedural advantages not fully captured in this analysis, such as reduced puncture force.

Despite these limitations, this study confirms that the novel tapered stylet design does not compromise diagnostic accuracy and offers a viable alternative to existing FNB options. Future studies should evaluate potential procedural advantages (e.g., reduced puncture force, fewer passes) and assess tissue suitability for downstream molecular applications.

## 5. Conclusions

In conclusion, the novel tapered stylet tip FNB needle demonstrated equivalent diagnostic accuracy and sensitivity compared to conventional end-cutting FNB needles in sampling solid lesions. While the new design may offer practical advantages in puncture mechanics, these did not translate into superior diagnostic outcomes in this study. As the clinical role of EUS expands into molecular diagnostics and precision medicine, future studies should assess not only diagnostic accuracy but also specimen adequacy for advanced testing, procedural efficiency, and operator experience. Continued innovation in needle technology remains essential to meet these evolving demands.

## Figures and Tables

**Table 1:** Demographic, clinical, and technical characteristics.

Variable	Acquire S (n = 129)	Controls (n = 141)	*p*-Value
Age, years, median (IQR)	69 (61.7–76)	68 (57–74.5)	0.287 *
Gender, n (%)			
Male	65 (50.4)	73 (51.8)	0.820 ^†^
Female	64 (49.6)	68 (48.2)	-
Site of lesion, n (%)			
Pancreas	95 (73.6)	94 (66.7)	0.211 ^†^
Lymph node	9 (7)	12 (8.5)	0.638 ^†^
Bile duct	7 (5.4)	18 (12.8)	**0.038 ^†^**
Liver	1 (0.8)	3 (2.1)	0.358 ^†^
Ampulla	5 (3.9)	4 (2.8)	0.635 ^†^
Other	12 (9.3)	10 (7.1)	0.507 ^†^
Lesion size, median (IQR)	25 (193–535)	30 (20–40)	0.023 *
Needle size, n (%)			
22ga	126 (97.7)	133 (94.3)	0.164 ^†^
25ga	3 (2.3)	8 (5.7)	-
Number of passes, mean (±SD)	2.89 (0.85)	2.76 (0.85)	0.158 ^‡^
Final diagnosis of malignancy, n (%)	103 (79.8)	102 (72.3)	0.15 ^†^
Malignancy subtypes, n (%)			
Adenocarcinoma	83 (80.6)	80 (78.4)	0.202 ^†^
Neuroendocrine tumour	15 (14.6)	9 (8.8)	0.13 ^†^
GIST	1 (1)	2 (2)	0.614 ^†^
Lymphoma	0 (0)	4 (3.9)	0.054 ^†^
Non-small cell lung cancer	0 (0)	2 (2)	0.175 ^†^
Leiomyosarcoma	0 (0)	1 (1)	0.338 ^†^
Metastatic melanoma	1 (1)	0 (0)	0.295 ^†^
Metastatic renal cell cancer	0 (0)	2 (2)	0.175 ^†^
Metastatic transitional cell cancer	1 (1)	0 (0)	0.318 ^†^
Hepatocellular carcinoma	1 (1)	1 (1)	0.95 ^†^
Squamous cell pancreatic cancer	1 (1)	0 (0)	0.295 ^†^
Acinar cell cancer	0 (0)	1 (1)	0.338 ^†^

* Mann–Whitney U test; ^†^ χ^2^ test; ^‡^ Student’s *t*-test; IQR, interquartile range; SD, standard deviation.

**Table 2:** Diagnostic performance for all pathologies.

Outcome	Acquire S		Controls		*p*-Value
	%	(95% CI)	%	(95% CI)	
Sensitivity	90.3	(82.9–95.2)	88.2	(80.3–93.8)	0.147 ^†^
Specificity	100	(86.8–100)	100	(91–100)	-
Accuracy	92.2	(86.2–96.2)	91.5	(85.6–95.5)	0.634 ^†^
Negative predictive value	72.2	(59.1–82.4)	76.5	(65.6–84.7)	0.82 ^†^
Positive predictive value	100	(96.1–100)	100	(96–100)	-

^†^ χ^2^ test.

**Table 3:** Diagnostic performance for all pathologies—strict reporting criteria.

Outcome	Acquire S		Controls		*p*-Value
	%	(95% CI)	%	(95% CI)	
Sensitivity	84.5	(76−90.85)	80.4	(71.35–87.59)	0.115 ^†^
Specificity	100	(86.8–100)	100	(90.97–100)	-
Accuracy	87.6	(80.64–92.74)	85.82	(78.95–91.12)	0.667 ^†^
Negative predictive value	61.9	(50.88–71.83)	66.1	(56.83–74.28)	0.667 ^†^
Positive predictive value	100	(95.85–100)	100	(95.6–100)	-

^†^ χ^2^ test.

**Table 4:** Diagnostic performance for pancreatic solid lesions.

Outcome	Acquire S		Controls		*p*-Value
	%	(95% CI)	%	(95% CI)	
Sensitivity	93.8	(86–97.9)	94.4	(86.4–98.5)	0.29 ^†^
Specificity	100	(78.2–100)	100	(84.6–100)	-
Accuracy	94.7	(88.1–98.3)	95.7	(89.5–98.8)	0.745 ^†^
Negative predictive value	75	(56.2–87.5)	84.6	(68–93.4)	0.187 ^†^
Positive predictive value	100	(95.2–100)	100	(94.7–100)	-

^†^ χ^2^ test.

## Data Availability

The data underlying this article will be shared upon reasonable request to the corresponding author. This manuscript, including related data, figures, and tables, has not been previously published and is not under consideration elsewhere.
